# The Metaproteomics Initiative: five years of community-driven progress

**DOI:** 10.1186/s40168-026-02463-0

**Published:** 2026-07-23

**Authors:** Tim Van Den Bossche, Lucia Grenga, Gelio Alves, Magnus Ø. Arntzen, Dirk Benndorf, Madita Brauer, Daniel Figeys, Celine Henry, Robert L. Hettich, Robert Heyer, Pratik D. Jagtap, Nico Jehmlich, Manuel Kleiner, Leyuan Li, Bart Mesuere, Martin Pabst, Jagroop Pandhal, Phillip B. Pope, Jana Seifert, Anke Trautwein-Schult, Pieter Verschaffelt, Paul Wilmes, Jean Armengaud, Benoit J. Kunath

**Affiliations:** 1 Department of Biomolecular Medicine, Faculty of Medicine and Health Sciences, Ghent University, Ghent, 9052 Belgium; 2 VIB-UGent Center for Medical Biotechnology, VIB, Ghent, 9052 Belgium; 3 Département Médicaments et Technologies pour la Santé (DMTS), Université Paris-Saclay, CEA, INRAE, SPI, Bagnols-sur-Cèze, France; 4 Division of Intramural Research, National Library of Medicine, National Institutes of Health, Bethesda, MD 20894 USA; 5 Faculty of Chemistry, Biotechnology and Food Science, Norwegian University of Life Sciences, Ås, Norway; 6 Anhalt University of Applied Sciences, Applied Biosciences and Process Engineering, Köthen, 06366 Germany; 7 Department of Life Sciences and Medicine, Faculty of Science, Technology and Medicine, University of Luxembourg, Esch-sur-Alzette, Luxembourg; 8 School of Pharmaceutical Sciences, Faculty of Medicine, University of Ottawa, Ottawa, ON Canada; 9 Quadram Institute Bioscience, Norwich Research Park, Norwich, Norfolk UK; 10 University of East Anglia, Norwich, Norfolk UK; 11 Université Paris-Saclay, INRAE, AgroParisTech, Micalis Institute, Jouy-en-Josas, 78350 France; 12 Biosciences Division, Oak Ridge National Laboratory, Oak Ridge, TN USA; 13 Multidimensional Omics Data Analyses group, Leibniz-Institut für Analytische Wissenschaften – ISAS – e.V., Bunsen-Kirchhoff-Straße 11, Dortmund, 44139 Germany; 14 Multidimensional Omics Data Analyses group, Faculty of Technology, Bielefeld University, Universitätsstraße 25, Bielefeld, 33615 Germany; 15 Department of Biochemistry, Molecular Biology, and Biophysics, University of Minnesota, Minneapolis, MN USA; 16 Department of Molecular Toxicology, Helmholtz-Centre for Environmental Research - UFZ GmbH, Permoserstrasse 15, Leipzig, 04318 Germany; 17 Department of Plant and Microbial Biology, North Carolina State University, Raleigh, NC USA; 18 State Key Laboratory of Medical Proteomics, National Center for Protein Sciences, Beijing, China; 19 Department of Mathematics, Computer Science, and Statistics, Ghent University, Ghent, Belgium; 20 Department of Biotechnology, Delft University of Technology, van der Maasweg 9, Delft, 2629HZ The Netherlands; 21 School of Chemical, Materials and Biological Engineering, Faculty of Engineering, University of Sheffield, Sheffield, UK; 22 Centre for Microbiome Research, Faculty of Health, School of Biomedical Sciences, Queensland University of Technology, Translational Research Institute, Woolloongabba, Australia; 23 Institute of Animal Science, University of Hohenheim, Stuttgart, Germany; 24 HoLMiR - Hohenheim Center for Livestock Microbiome Research, University of Hohenheim, Stuttgart, Germany; 25 Department of Microbial Proteomics, University of Greifswald, Institute of Microbiology, Greifswald, 17489 Germany; 26 Luxembourg Centre for Systems Biomedicine, University of Luxembourg, Esch-sur-Alzette, Luxembourg; 27 Department of Life Sciences and Medicine, Faculty of Science, Technology and Medicine, University of Luxembourg, Esch-sur-Alzette, Luxembourg; 28 Bioinformatics and Artificial Intelligence, Department of Medical Informatics, Luxembourg Institute of Health (LIH), Strassen, 1445 Luxembourg

## Abstract

The Metaproteomics Initiative was officially launched in 2021 to strengthen collaboration, promote knowledge exchange, and support and lead standardization efforts within the growing metaproteomics community. Over the past 5 years, the Initiative has developed into a structured, global network of researchers. It has launched community-driven benchmark studies, helped shape emerging metadata and reporting standards, developed practical guidance and training materials, organized international symposia, and fostered connections across the microbiome research landscape (https://metaproteomics.org/). We outline the Initiative’s organization, activities, achievements, and ongoing efforts, and reflect on how sustained, community-led coordination has shaped the development of metaproteomics as a field. We further position the Grand Metaproteome Challenges as a next step toward coordinated, community-scale biological research, aimed at advancing functional microbiome studies across clinical, industrial, and environmental application domains, and invite engagement from the wider microbiome and omics communities.

Video Abstract

**Supplementary Information:**

The online version contains supplementary material available at 10.1186/s40168-026-02463-0.

## Introduction

Microbiomes underpin the functioning of every ecosystem, playing essential roles in processes ranging from nutrient cycling to host-microbe interactions and health. Understanding how microbiomes operate under specific environmental or host-associated conditions requires tools that move beyond taxonomic inventories to capture molecular activity [1, 2]. Metaproteomics offers precisely this functional lens, providing a direct readout of the proteins actively produced within the microbiomes and their environments. By quantifying these proteins in their context-dependent expression and linking them to their taxonomic origins and functional roles, metaproteomics bridges the gap between genomic potential and phenotypic expression. In doing so, it enables researchers to identify the functions expressed by microbes from all domains of life simultaneously and their regulations across space and time under differing conditions, while also capturing post-translational modifications that further refine functional interpretation [3–7]. This capacity to generate functional insights with taxonomic resolution makes metaproteomics a powerful complement to metagenomics, metatranscriptomics, and metabolomics, and therefore a central component of multi-omics microbiome research [8]. Recent studies exemplify the biological potential that improved metaproteomics methods can unlock: from mapping gut microbiome functional responses to drugs and diet [9, 10], to uncovering host–microbiome interaction networks relevant to intestinal disease [11], and forecasting microbial community dynamics using multi-omics integration [12]. Realizing this potential at scale, however, requires the kind of methodological harmonization, reproducible workflows, and shared standards that the field is working to establish.

As the field matured, it became increasingly clear that advancing metaproteomics requires more than individual efforts or isolated case studies. The complexity of microbial ecosystems, the diversity of experimental designs, and the rapid evolution of different analytical tools and bioinformatics workflows called for coordinated community-wide actions [13]. Among the most persistent methodological challenges are protein inference ambiguity, whereby shared peptides prevent unambiguous assignment of identified peptides to specific proteins or organisms [14]; the dependence on reference databases that remain incomplete or taxonomically biased for many microbial ecosystems [15, 16]; and variability in peptide and protein quantification across analytical workflows and laboratories [17]. These limitations directly affect the reproducibility, comparability, and biological interpretability of metaproteomics results, and addressing them in a coordinated manner requires community-wide effort. Approximately five years ago, a growing number of researchers recognized that the full potential of metaproteomics could only be realized through collective effort. What began as a shared recognition of an opportunity soon matured into a structured effort to connect researchers, share discoveries, standardize methodologies, and lower entry barriers for new users. Building on discussions that emerged during the early International Metaproteomics Symposia (IMS, 2016–2018) and subsequent community efforts, the Metaproteomics Initiative (MPI) was established in 2021 to consolidate these activities into a cohesive community framework. Five years on, this commentary provides an overview of the Initiative’s development, key activities, and ongoing efforts, and outlines directions for the next phase of community-driven metaproteomics.

## Five years of MPI built on ten years of IMS and twenty years of metaproteomics

The first metaproteomic studies originated in 2005, marking the beginning of a field focused on characterizing the functional output of microbial communities through large-scale protein identification [18, 19]. Over the past two decades, metaproteomics has evolved from a niche methodology into a recognized discipline with demonstrated applications in microbial ecology, host-microbiome interactions, human health, and biotechnology. Despite this growing body of research, metaproteomics efforts were largely fragmented across communities, experimental designs, and bioinformatic workflows.

**Fig. 1:**
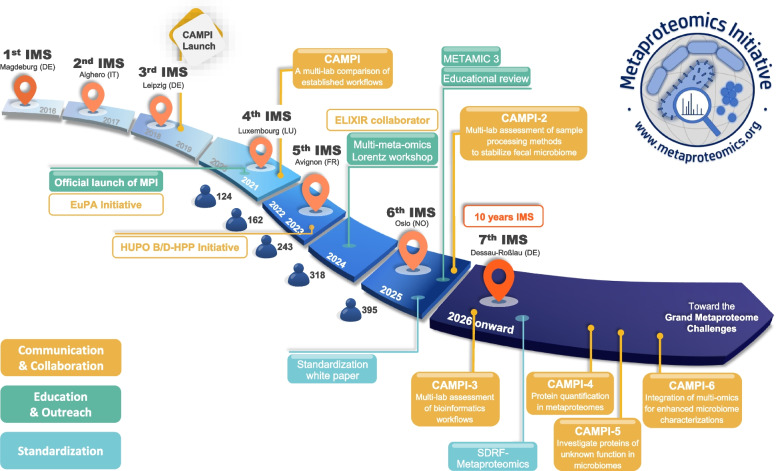
Roadmap of the Metaproteomics Initiative, illustrating its evolution from early community efforts and the first International Metaproteomics Symposia towards coordinated community activities, benchmark studies, and standardization efforts. Events and milestones are color-coded according to the three pillars of the Initiative. Member growth is indicated by the silhouettes

To address this fragmentation, the IMS were established in 2016 as a recurring forum for sharing progress, identifying bottlenecks, and fostering momentum towards coordinated community initiatives. A turning point came in 2018 with the launch of the Critical Assessment of MetaProteome Investigation (CAMPI) benchmarking study at the 3rd IMS in Leipzig, Germany. This effort introduced the first large-scale, community-driven benchmarking study in metaproteomics, making methodological variability explicit and catalyzing broader discussions that would ultimately lead to the formation of the MPI (Fig. 1).

In 2021, the Initiative was officially recognized as a EuPA Initiative, providing formal support and visibility. This was followed by HUPO’s endorsement in 2023 and the start of collaborations with the ELIXIR Microbiome Community in 2024 [20]. Together, these developments positioned the MPI as a bridge between early, scattered efforts and the coordinated development of a global metaproteomics community. This growth in membership and reach is documented in Supplementary Fig. 1.

## Founding vision and organizational structure

The Metaproteomics Initiative was established as a grassroots effort to address a growing need for structured collaboration, accessible knowledge exchange, and greater methodological consistency within the field. Rather than being initiated by a single institution, it emerged organically from the metaproteomics research community through open discussions and recurring meetings.

From its inception, the Initiative has functioned as a coordination platform, bringing together microbiologists, experimentalists, bioinformaticians, and tool developers. Its mission is to promote the dissemination of metaproteomics’ fundamentals, advancements, and applications through collaborative networking in microbiome research. It serves as the central information hub and open meeting place where newcomers and experts interact to exchange, standardize, and accelerate experimental and bioinformatics methodologies in the field. This is achieved through our website (www.metaproteomics.org), presentations, online communication channels, collaborative projects, and symposia.

The Initiative is governed by an elected Executive Board (EB), which is responsible for coordinating activities and ensuring the smooth operation of the organization [21]. The current EB, elected until the end of 2026, with new elections for the subsequent 3-year term to be held at the next IMS in June, includes two administrators who lead the Initiative and its EB, one Secretary, one Communication Lead, one Education Lead, one Webmaster, one Early Career Representative, past and incoming Symposium Organizers, and six Scientific Advisors. The Scientific Advisors form a dedicated Scientific Committee that provides long-term vision.

Executive Board meetings are held monthly and are open to all members of the Initiative. All researchers in the community are welcome to join the Initiative without obligations or fees, and members may propose new Working Groups (WGs) to address time-bound tasks aligned with the mission of the Initiative. These WGs are flexible, task-oriented subgroups with clearly defined end goals and are open to participation by any member. Examples include benchmarking efforts such as the CAMPI series [22, 23], the development of the educational review [24], or standardization efforts [25].

It is worth noting that long-term sustainability is an important consideration for all community-driven initiatives, including the Metaproteomics Initiative. While the Initiative was formally launched in 2021, it builds on a broader metaproteomics community that has organized symposia and collaborative activities for several years. The Initiative relies largely on voluntary contributions from researchers, elected leadership, working groups, and support through scientific societies. This model requires sustained engagement, but also enables flexible collaboration, low barriers to participation, and shared ownership of community activities.

**Fig. 2:**
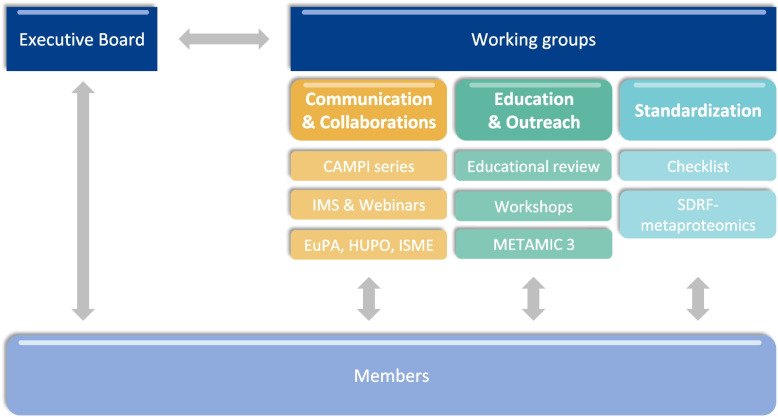
Organigram of the Metaproteomics Initiative, highlighting the three pillars of the Initiative (Communication and Collaboration, Education and Outreach, and Standardization) and the different levels of interactions within the Initiative. Adapted from Metaproteomics Initiative’s organizational structure [21]

This open, inclusive structure, formalized in the Initiative’s governing document [21] ensures that the Initiative remains community-led and aligned with the evolving needs of the global metaproteomics community (Fig. 2).

## Milestones and ongoing work across the three pillars

### Communication and collaboration

The Initiative has played a central role in strengthening international collaboration and fostering a sense of community across the growing metaproteomics field (Supplementary Fig. 1). The IMS series has continued under its coordination, with three editions held between 2021 and 2025, and the next already scheduled for Dessau-Roßlau in 2026. To maintain momentum between symposia, the Initiative hosts several webinars that support ongoing community engagement.

These meetings bring together key speakers, established researchers, and early-career researchers to share their work and insights. Participants are encouraged to present preliminary or unpublished results, fostering an open environment, early feedback and collaboration. Programs typically combine scientific presentations with roundtable sessions, guided discussions, and dedicated hands-on training sessions for students and newcomers. The IMS meetings therefore serve as important venues for exchanging knowledge, presenting novel findings, initiating new projects, and shaping the field’s direction.

In addition to these meetings, the Initiative supports year-round communication through social media presence and several newsletters per year. It maintains close interaction with EuPA and HUPO [26], and collaborates with the ELIXIR Microbiome Community [20] and the Genomics Standards Consortium (GSC) to promote cross-omics integration and share best practices. Through these connections, metaproteomics is embedded in broader microbiome research discussions and represented in multi-omics standardization efforts. Whereas HUPO focuses primarily on human proteomics and the GSC and ELIXIR Microbiome Community address genomic data standards and broader bioinformatics infrastructure, MPI is uniquely dedicated to proteins synthesized by microbial communities regardless of their environmental or host context, a focus that makes it a natural bridge between the proteomics and microbiome research worlds, and an excellent complementary role and added value within the broader omics landscape.

Community-driven benchmarking represents a further core element of collaboration within the Initiative. The CAMPI series exemplifies how community-driven studies can build consensus, generate reusable datasets, and compare analytical pipelines across laboratories and platforms. The following CAMPI studies have been completed:

- CAMPI(−1) [22] focused on evaluating the diversity in sample preparation, mass spectrometry (MS) workflows, and bioinformatics pipelines using a simplified gut model and a fecal sample. It demonstrated that while peptide-level differences exist across workflows, functional profiles remain robust. The dataset is publicly available through ProteomeXchange/PRIDE under accession PXD023217 and ranks among the top 4% most downloaded PRIDE datasets at the time of writing, illustrating continued community interest in this benchmarking resource.
- CAMPI-2 [23] assessed five stabilization methods for fecal samples during transport and storage at room temperature, identifying a commercial swab-based device as the most reproducible across several labs. This newer dataset is also publicly available through ProteomeXchange/PRIDE under accession PXD060335 and ranks already among the top 21% most downloaded PRIDE datasets at the time of writing.
- CAMPI-3 (*manuscript in preparation*) investigates the impact of different software tools and protein inference strategies on metaproteomics results, aiming to assess reproducibility in protein-level identification and accuracy in taxonomic identification across workflows.

Several upcoming studies are in preparation:

- CAMPI-4 - Quantification: will assess reproducibility and response linearity of peptide/protein quantification using data-independent acquisition (DIA-MS and defined spike-ins across labs.
- CAMPI-5 - PUF: will mine metaproteomics data to detect Proteins of Unknown Function (PUFs), which are conserved but unannotated proteins, and propose strategies to improve their functional characterization.
- CAMPI-6 - Multi-omics: aims to benchmark multi-omics pipelines using existing and new controlled datasets to establish best practices.

Collectively, these benchmarking efforts provide a practical framework for cross-laboratory collaboration, support method optimization, and contribute to the development of community-aligned protocols and shared resources for both the metaproteomic field and other microbiome-related communities. It should be noted, however, that harmonization of experimental and computational methodologies remains an ongoing process. Variability across laboratories and workflows persists, and the adoption of community-endorsed standards is still incomplete across the field. Consensus-driven approaches, while a powerful mechanism for coordinating progress, do not fully eliminate methodological heterogeneity. Addressing these limitations is precisely what motivates the continued expansion of the CAMPI series and the Initiative’s growing engagement with broader microbiome and omics communities, working together toward more consistent, interoperable, and widely adopted practices.

### Education and outreach

Education and outreach constitute a central pillar of the Metaproteomics Initiative, aimed at supporting both newcomers to the field and researchers seeking to deepen their expertise. As a cornerstone of these efforts, the Initiative developed and published “The microbiologist’s guide to metaproteomics” [24], an open-access review that provides a structured entry point into the field. This guide covers experimental design, sample preparation, MS strategies, and data analysis workflows, and has been widely used to support the training of microbiologists who are new to metaproteomics.

Beyond this guide, members of the Initiative regularly represent the community at major mass spectrometry, proteomics, and microbiology conferences, where they (co-)organize workshops, training sessions, and technical lectures. By providing accessible educational content and maintaining a strong presence at international venues, the Initiative lowers barriers to entry and promotes good practices within the community.

In addition, the Initiative organized a more dedicated event, the Deciphering Microbiome Functions workshop held in March 2024 (https://www.lorentzcenter.nl/deciphering-microbiome-functions.html), which brought together the microbiome community for a week of interaction and collaboration. The workshop addressed challenges related to data interoperability, standardization, and accessibility across meta-omics fields, and explicitly aimed to improve the integration of multi-omics approaches for more comprehensive insights into microbial functions and interactions.

More recently, education and training efforts within the Initiative have expanded to include formal doctoral training through the launch of a Marie Skłodowska-Curie doctoral network (METAMIC 3, https://metamic3.isas.de/). This doctoral network focuses on microbiome-based interventions for health, agriculture, and environmental sustainability within a One Health framework, building on advanced metaproteomics methodologies and fundamental insights into microbiome function [27]. METAMIC 3 officially began with its kickoff meeting in October 2025, marking the start of a new phase of coordinated, international doctoral research embedded within the Initiative.

### Standardization

Standardization constitutes the third strategic pillar of the Metaproteomics Initiative, although focused activities in this area have intensified primarily over the past few years. As metaproteomics has expanded in scale and diversity, differences in experimental design, data processing, and reporting practices have increasingly limited interoperability, reproducibility, and cross-study reuse. Similar challenges related to insufficient and inconsistent metadata annotation have been highlighted more broadly in the proteomics field, underscoring the central role of structured metadata in enabling large-scale data reuse and interpretation [28]. Recognizing both this broader context and the growing bottleneck within metaproteomics, the Initiative has placed increasing emphasis on coordinated standardization efforts to support transparent interpretation and data reuse across metaproteomics studies.

First, the Initiative authored a community-developed perspective paper on standardization [25]. This manuscript introduces a reporting checklist tailored specifically to metaproteomics and outlines ongoing efforts to capture essential metadata in a structured way, drawing on established standards from proteomics and microbiome research. These contributions help align the field with FAIR principles and facilitate transparent, reproducible science.

In parallel, the Initiative is developing Sample and Data Relationship Format (SDRF)-Proteomics templates for metaproteomics. SDRF-Proteomics is a tab-delimited file format that enables structured annotation of sample metadata and their relationship to data files in proteomics experiments [29]. These metaproteomics templates will incorporate relevant MIxS environmental extensions from the GSC [30], and will therefore describe not only proteomics-specific metadata but also relevant microbial and environmental metadata, while supporting future interoperability and integration with other omics layers.

Through these efforts, the Metaproteomics Initiative provides both practical infrastructure and strategic vision to support robust and coordinated progress in the microbiome field.

## Looking ahead: preparing for global large-scale projects

The collective efforts in collaboration, education, and standardization described above have strengthened the expertise, methodological foundation, and coordination capacity of the metaproteomics community. Building on these accomplishments and on the uniquely collaborative culture of the field, the community is increasingly positioned to move beyond isolated studies towards coordinated research programs that address biological questions exceeding the scope of any single laboratory.

These efforts are ultimately driven by concrete biological and clinical questions: “how do microbial communities respond to disease, medication, or environmental change at the functional level, and how can this knowledge be translated into actionable insights for health, agriculture, and ecosystem management?”. To catalyze this next step, we propose a series of coordinated, community-scale research projects referred to as “Grand Metaproteome Challenges”. These projects are presented here as early-stage directions and an open invitation for the community to engage, shape, and co-develop these efforts together. They are envisioned as multi-institutional efforts aimed at addressing major biological questions through large-scale, harmonized studies across diverse application domains. Potential examples include coordinated efforts to globally map the functional diversity of the microbiome across human health, agriculture, and environmental systems, the generation of an annotated Human Metapeptidome Atlas, or a Microbiome System Project to determine functions, networks and interactions in microbiomes such as in climate-relevant ecosystem studies, or metaproteomics-based surveillance of antimicrobial resistance.

The CAMPI series provides essential groundwork for enabling such projects. By generating openly available reference datasets and benchmarks, systematically evaluating critical qualitative and quantitative performance metrics, and supporting the harmonization of experimental and computational methodologies, CAMPI establishes the technical and conceptual basis required for reliable large-scale collaboration. In this sense, CAMPI focuses on best practices and provides the technical and conceptual foundation upon which large-scale community projects can be built.

The proposed Grand Metaproteome Challenges would extend this foundation by applying validated protocols and harmonized workflows at scale. Rather than benchmarking methods, these projects would leverage agreed-upon standards to address biological questions through coordinated data generation and analysis. Such efforts could accelerate the maturation of metaproteomics as a core functional layer within microbiome research and demonstrate its value in addressing questions that require structured, international collaboration across clinical, industrial, and environmental contexts.

Realizing this vision will require continued strengthening of cross-community alignment. Interoperability across meta-omics disciplines and application domains remains essential for meaningful integration. Closer coordination with communities such as HUPO-PSI, the ELIXIR Microbiome Community, and the GSC [20, 26, 31] will help align (meta)data standards, reporting guidelines, and data exchange formats. In parallel, deeper integration with other meta-omics fields can support coherent multi-omics study designs through coordinated community efforts, including the CAMPI-6 multi-omics benchmarking study and cross-disciplinary workshops, as well as initiatives such as the Critical Assessment of Metagenome Interpretation (CAMI) challenges [32], thereby enabling integrated interpretation of microbiome structure and function. Where CAMI benchmarks sequence-based metagenomic tools, CAMPI addresses the same need at the protein level, making the two efforts complementary across the central dogma, together spanning from genomic potential to functional activities.

The Metaproteomics Initiative is well positioned to coordinate these preparatory activities by bringing together stakeholders, curating shared resources and reference datasets, aligning standards and evaluation criteria, and ultimately mobilizing the community around this next phase of metaproteomics.

## Conclusion

Over the past five years, the Metaproteomics Initiative has matured into a vibrant and active global community, structured around three strategic pillars: communication and collaboration, education and outreach, and standardization. Through symposia, community-driven benchmarking efforts, educational resources, and standardization activities, the Initiative has contributed to both the scientific and organizational foundations of metaproteomics.

This Commentary documents how sustained, community-led coordination can help address persistent challenges in a rapidly evolving field, including methodological fragmentation, limited interoperability, and barriers to data reuse. Looking ahead, the continued maturation of the metaproteomics community enables a shift from primarily fragmented, method-centric evaluation toward more coordinated, community-scale biological research efforts. In this context, the Grand Metaproteome Challenges outlined here represent a natural next step: building on insights from benchmarking studies such as CAMPI, shared standards, and collective expertise to address major biological questions that exceed the scope of individual laboratories, and to strengthen the contribution of functional protein-level information within microbiome research across clinical, industrial, and environmental contexts.

The Metaproteomics Initiative remains open and community driven. Researchers are invited to contribute by participating in upcoming CAMPI studies and future Grand Metaproteome Challenges, engaging in standardization and training activities, or standing as candidates in upcoming Executive Board elections. We also welcome researchers from adjacent disciplines who see value in this approach and are interested in engaging with, or adapting, similar grassroots initiatives in their own fields. These discussions and collaborations will continue at the next International Metaproteomics Symposium, to be held from 21 to 24 June 2026 in Dessau-Roßlau, Germany (https://metaproteomics.org/symposia/seventh/).

## Supplementary Information

Supplementary Material 1: Supplementary Fig. 1. Growth and Impact of the Metaproteomics Initiative. The upper panel displays the continuous growth of members and participating research groups of the metaproteomics since its launch in 2021. The lower panel shows the cumulative number of publications produced under the Initiative’s three pillars alongside the growth in citations over time, demonstrating both output and uptake by the community.

## Data Availability

No datasets were generated or analysed during the current study.
