# Supplementary material for: The Metaproteomics Initiative: five years of community-driven progress
Source: Microbiome. 2026 Jul 23;14:186. doi: 10.1186/s40168-026-02463-0 (PMC13393725; doi:10.1186/s40168-026-02463-0)
Supplement: Supplementary file 2 — Supplementary Material 1: Supplementary Fig. 1. Growth and Impact of the Metaproteomics Initiative. The upper panel displays the continuous growth of members and participating research groups of the metaproteomics since its launch in 2021. The lower panel shows the cumulative number of publications produced under the Initiative’s three pillars alongside the growth in citations over time, demonstrating both output and uptake by the community. [file 40168_2026_2463_MOESM1_ESM.docx]

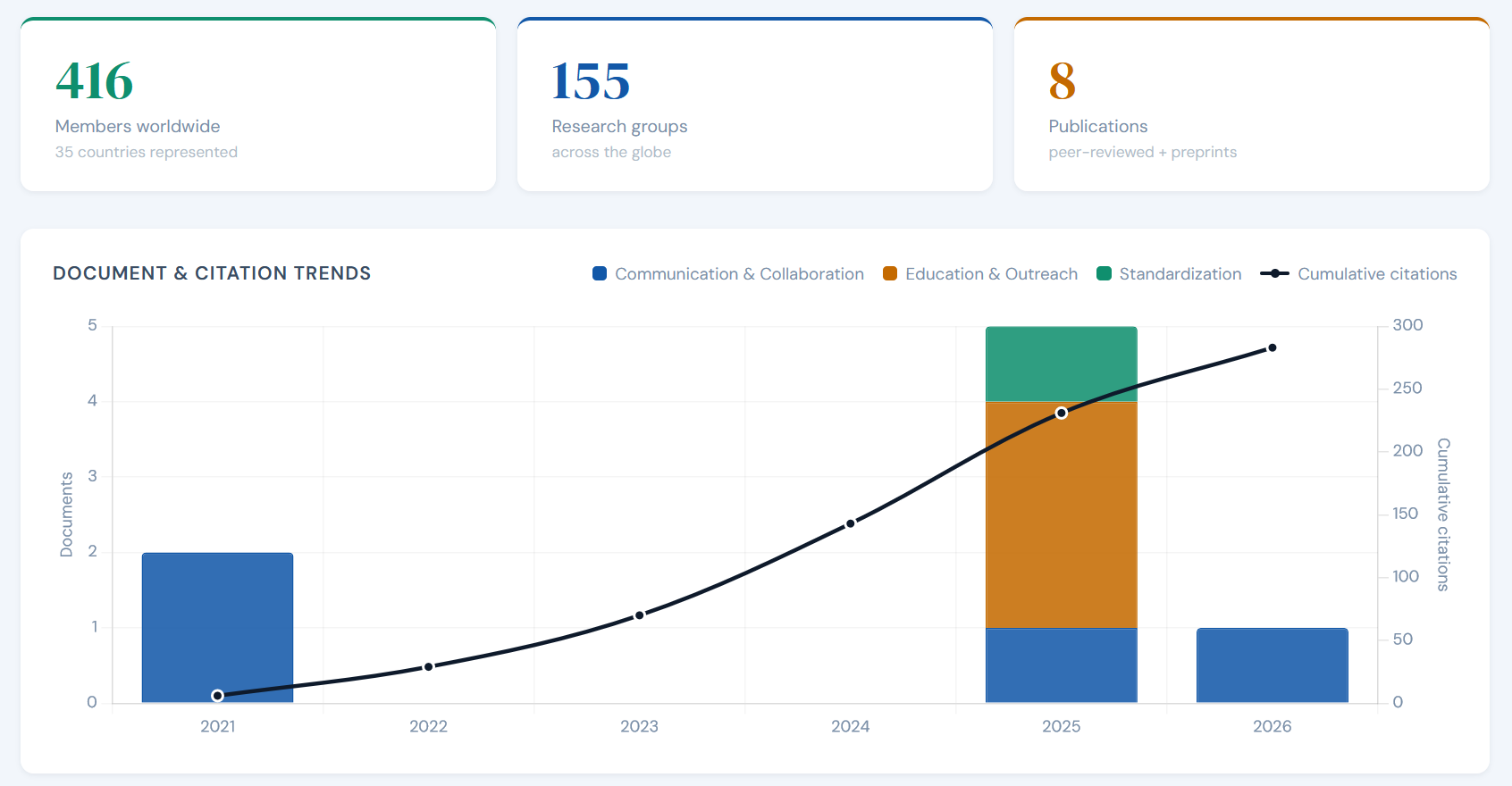

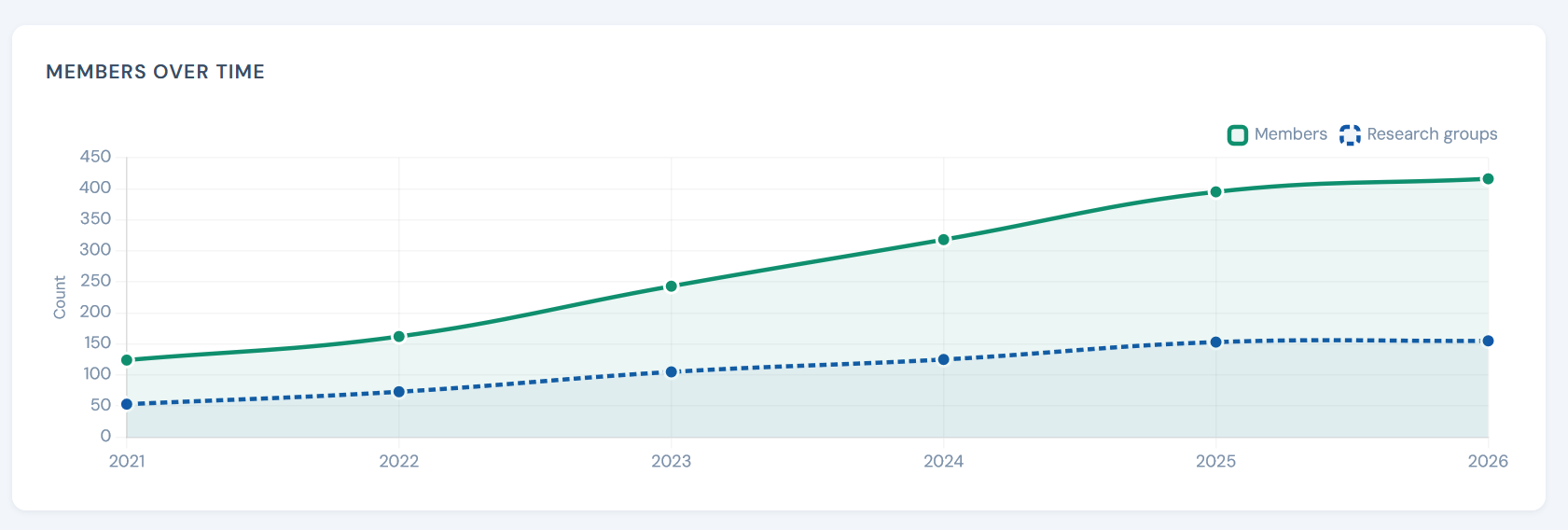

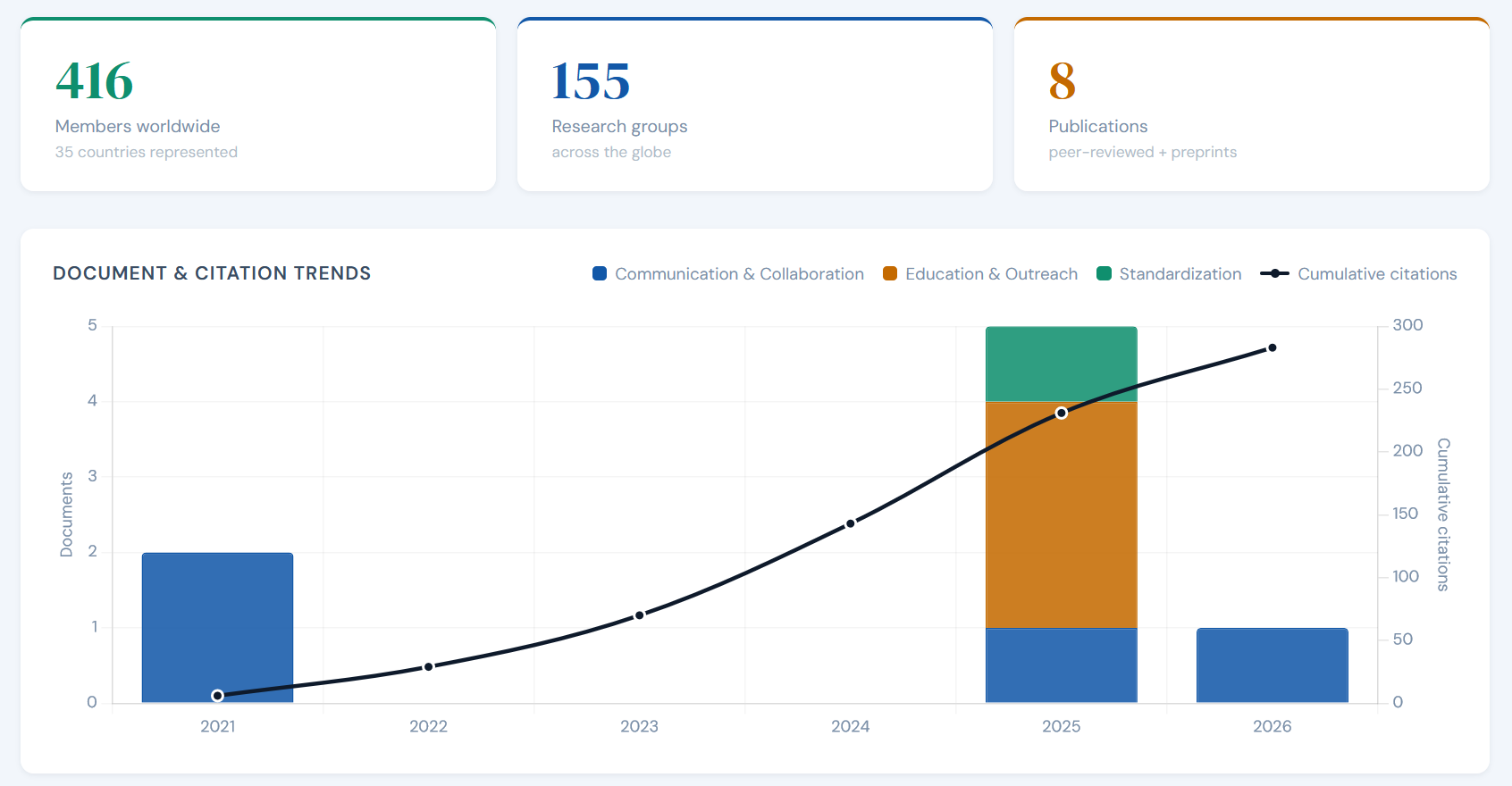


**Supplementary Figure 1. Growth and Impact of the Metaproteomics Initiative.** The upper panel displays the continuous growth of members and participating research groups of the metaproteomics since its launch in 2021. The lower panel shows the cumulative number of publications produced under the Initiative's three pillars alongside the growth in citations over time, demonstrating both output and uptake by the community.
